# Role of the NLRP3 Inflammasome in Preeclampsia

**DOI:** 10.3389/fendo.2020.00080

**Published:** 2020-02-25

**Authors:** Koumei Shirasuna, Tadayoshi Karasawa, Masafumi Takahashi

**Affiliations:** ^1^Department of Animal Science, Tokyo University of Agriculture, Atsugi, Japan; ^2^Division of Inflammation Research, Center for Molecular Medicine, Jichi Medical University, Shimotsuke, Japan

**Keywords:** NLRP3 inflammasome, pregnancy, preeclampsia, interleukin-1β, inflammation

## Abstract

Reproduction involves tightly regulated series of events and the immune system is involved in an array of reproductive processes. Disruption of well-controlled immune functions leads to infertility, placental inflammation, and numerous pregnancy complications, including preeclampsia (PE). Inflammasomes are involved in the process of pathogen clearance and sterile inflammation. They are large multi-protein complexes that are located in the cytosol and play key roles in the production of the pivotal inflammatory cytokines, interleukin (IL)-1β and IL-18, and pyroptosis. The nucleotide-binding oligomerization domain, leucine-rich repeat-, and pyrin domain-containing 3 (NLRP3) inflammasome is a key mediator of sterile inflammation induced by various types of damage-associated molecular patterns (DAMPs). Recent evidence indicates that the NLRP3 inflammasome is involved in pregnancy dysfunction, including PE. Many DAMPs (uric acid, palmitic acid, high-mobility group box 1, advanced glycation end products, extracellular vesicles, cell-free DNA, and free fatty acids) are increased and associated with pregnancy complications, especially PE. This review focuses on the role of the NLRP3 inflammasome in the pathophysiology of PE.

## Introduction

Reproduction, including development of oocyte and sperm, ovulation, corpus luteum function, fertilization, implantation, placentation, maintenance of pregnancy, and parturition, is essential for species maintenance, and reproductive events for next generation are tightly regulated (1). Pregnancy has been studied extensively over the years (2). From the perspective of the maternal immune system, a conceptus is a semi-allogeneic tissue that must be rejected; however, that does not generally happen. It was quickly ruled out that the fetus is shielded from the maternal immune system via the placenta acting as a physical barrier because the fetal extravillous trophoblast cells deeply penetrate the uterine mucosa and directly communicate with various maternal immune cells to avoid rejection (3).

Inflammation is basically a complex protective immune response to harmful stimuli such as pathogens, damaged or dead cells, and irritants (4). This response is tightly regulated by the host, enabling survival after infection or injury and maintaining tissue homeostasis. However, excessive inflammation may cause chronic or systemic inflammatory diseases. On the other hand, the immune system also contributes to the regulation of reproductive function and pregnancy (5). Immune-mediated processes such as tissue growth, remodeling, and differentiation are crucial to maintain pregnancy (1, 5). Disruption of well-controlled immune functions leads to infertility, placental inflammation, and numerous pregnancy complications, such as preeclampsia (PE), obesity during pregnancy, gestational diabetes mellitus (GDM), spontaneous abortion, and recurrent pregnancy loss (6–8).

**Figure 1:**
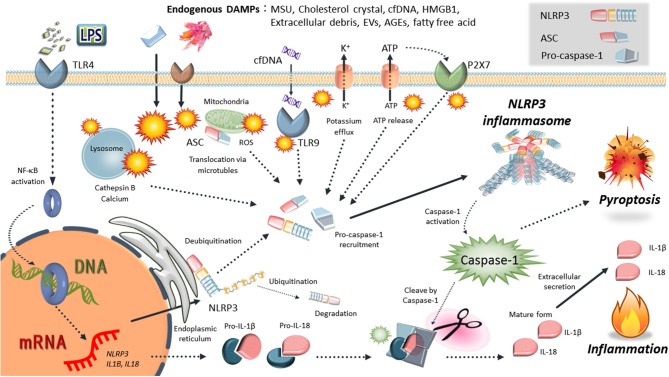
Schematic mechanisms of NLRP3 inflammasome activation. NLRP3 is activated by various endogenous DAMPs: uric acid crystals (MSU), cholesterol crustal, cell-free DNA (cfDNA), high-mobility group box 1 (HMGB1), extracellular debris, extracellular vesicles (EVs), advanced glycation end-products (AGEs), and free fatty acid. Various events such as intracellular ATP release, NLRP3 deubiqutination, relocalization, reactive oxygen species (ROS) generation, mitochondrial dysfunction, lysosome rapture, and cathepsin release occur depending on the effects of damage-associated molecular patterns (DAMPs). Then, inflammasome components, including NLRP3, ASC, and procaspase-1, form the NLRP3 inflammasome complexes. Finally, activated caspase-1 induces the inflammatory form of cell death known as pyroptosis and cleaves the precursor cytokines pro-IL-1β and pro-IL-18, generating the biologically active cytokines IL-1β and IL-18.

There is an increasing body of evidence to suggest that inflammation and immune cells are involved in both physiology and pathophysiology of pregnancy. Since infection is not involved in the majority of the phenomena related to pregnancy physiology and pathology, it remains unclear why inflammation is involved. Recently, there have been numerous reports of inflammasome mechanisms that control sterile inflammation involved in pregnancy pathologies. Inflammasomes are large multi-protein complexes found in the cytosol that play key roles in the production of the pivotal inflammatory cytokines, interleukin (IL)-1β and IL-18, and pyroptosis (inflammatory cell death) [(9–11); Figure 1]. In particular, nucleotide-binding oligomerization domain, leucine-rich repeat-, and pyrin domain-containing 3 (NLRP3) inflammasome is a key mediator of sterile inflammation. Excessive activation of the NLRP3 inflammasome contributes to the pathogenesis of a wide variety of diseases, such as diabetes, atherosclerosis, and obesity-induced insulin resistance (12–17). The present review focuses on the role of the NLRP3 inflammasome in placental inflammation and pregnancy complications, especially PE.

## Immune Cells Involved in Pregnancy

The most important immune cells that induce pregnancy immune tolerance is CD4+ regulatory T cells (Tregs) (18). The transcription factor, forkhead boxP3 (Foxp3), is a master regulator of the development and function of Tregs (19). The frequency of Foxp3+Tregs increases during normal pregnancy in the decidua and peripheral blood in humans and mice (20–22). Shima et al. (23) used an animal model to demonstrate that CD4^+^CD25^+^Foxp3^+^ Tregs play a critical role in regulating immune tolerance at the implantation site to support implantation and successful pregnancy. The frequency of Tregs is lower in human pregnancy complications such as PE or miscarriage (24). In addition, seminal fluid induces and accumulates paternal-specific Tregs that are involved in the preimplantation uterus, and insufficient expansion of Tregs against paternal antigens may trigger spontaneous abortion (25).

Natural killer (NK) cells, particularly decidual NK cells, are also essential immune cells involved in establishing pregnancy; they are the most abundant leukocyte population during the first trimester of human pregnancy (1, 26). Decidual NK cells directly communicate with extravillous trophoblast cells and other immune cells in the fetal-maternal boundary area, and promote fetal tolerance and pregnancy progression (26).

Monocytes also accumulate in the decidua, in a process that involves communication with trophoblast cells (1, 27). They can differentiate into dendritic cells (DCs) in the decidua during murine and human pregnancy (28, 29). DCs regulate immune tolerance by inducing effector T cell apoptosis and expansion of Tregs due to reduced antigen presentation, reduced expression of co-stimulatory molecules, or enhanced production of anti-inflammatory IL-10 (1, 30). Monocytes also differentiate into macrophages depending on the tissue, and polarization of macrophages is well-understood (inflammatory M1 and anti-inflammatory M2 type macrophages). It has been suggested that dysfunction of decidual macrophages and dysregulation of M1/M2 balance are critical events in the pathogenesis of PE. Moreover, activation of NLRP3 inflammasome in the reproductive organs including placenta is known to occur by these macrophages.

## Mechanisms Of NLRP3 Inflammasome Activation

Inflammasomes recognize various inflammation-inducing stimuli, such as endogenous danger/damage-associated molecular patterns (DAMPs) and exogenous pathogen-associated molecular patterns (PAMPs). They tightly regulate the production of proinflammatory cytokines such as IL-1β and IL-18 (9, 13, 31). The NLRP3 inflammasome is the most widely studied and is activated in response to a wide array of stimuli, including exogenous and endogenous danger signals [(9, 11); Figure 1]. The NLRP3 inflammasome is typically composed of NLRP3, apoptosis-associated speck-like protein containing a caspase recruitment domain (ASC), and caspase-1 as an IL-1β-converting enzyme (32). Activation of NLRP3 in response to danger signals leads to nucleation of ASC into prion-like filaments via pyrin domain (PYD)–PYD interactions (33). ASC is then linearly ubiquitinated for NLRP3 inflammasome assembly, followed by procaspase-1 interaction with ASC using caspase recruitment domain (CARD)-CARD interactions, forming its own prion-like filaments (34). Activated caspase-1 (a cysteine protease) cleaves the precursor cytokines, pro-IL-1β and pro-IL-18, generating the biologically active cytokines, IL-1β and IL-18, respectively (9–11). Moreover, active caspase-1 is able to induce pyroptosis as an inflammatory form of cell death due to cleaved gasdermin D (GSDMD) (35, 36). Caspase-1 proteolytically cleaves GSDMD into a N-terminal domain and C-terminal domain. Cleaved N-terminal domain of GSDMD binds to phosphatidylinositol phosphates and phosphatidylserine in the cell membrane, forming a 10–20 nm pore and induces a lytic form of cell death, pyroptosis (36). Another feature of gasdermin D-dependent pyroptosis is the release of IL-1β and IL-18 via GSDMD-forming cell membrane pore.

The production and secretion of mature IL-1β are regulated via two steps, including the transcription of pro-IL-1β and proteolytic processing into a mature form IL-1β by inflammasomes (9–11). Prior to its activation, NLRP3 must be primed in most cell types. Nuclear factor κB (NF-κB)-activating stimuli, such as lipopolysaccharide (LPS), upregulate mRNA expression of *NLRP3* and *IL-1*β, resulting in elevated expression of NLRP3 and pro-IL-1β protein (9–11). On the other hand, another priming step facilitates the rapid induction of the NLRP3 inflammasome via deubiquitination of NLRP3 (37, 38).

The upstream mechanisms of NLRP3 activation have been elucidated by many studies, and include the release of cathepsins into the cytosol after lysosomal destabilization, potassium efflux, generation of mitochondrial reactive oxygen species (ROS), and release of mitochondrial DNA (39, 40). Cytosolic leakage of cathepsin B via lysosomal rupture is essential for NLRP3 inflammasome activation, especially by endogenous DAMPs (41). Leakage of cathepsin B also leads to potassium efflux and mitochondrial damage. Potassium efflux and reduced potassium concentration within cells result in NLRP3 inflammasome activation (10). In response to potassium efflux, NEK7 (a member of the family of mammalian NIMA-related kinases) directly interacts with NLRP3 inflammasome (42, 43). Cellular and mitochondrial ROS production also act as NLRP3 inflammasome activators (44, 45). Furthermore, recent studies have demonstrated that the NLRP3 inflammasome is tightly regulated by multiple mechanisms, including ubiquitination, phosphorylation, nitrosylation, microRNAs, and endogenous regulators (e.g., pyrin-only proteins and CARD-only proteins) (9, 46–48).

Following NLRP3 activation through the above mentioned regulatory mechanisms, NLRP3 relocates from endoplasmic reticulum to the mitochondria, where it forms complexes with ASC (49). IL-1β and IL-18 secretion is regulated by caspase-1 activation by many NLRP3 inflammasome activators, including monosodium urate (MSU) crystals, silica crystals, asbestos, and cholesterol crystals (12, 13, 31, 50). Additionally to the canonical pathway of the NLRP3 inflammasome, the inflammasome activation can also be indirectly triggered by caspase-11 in mice (or the homologs caspase-4 and caspase-5 in humans), which has been termed the non-canonical inflammasome pathway (51). In this non-canonical pathway, caspase-11 directly recognized and binds to intracellular LPS, resulting in its oligomerization and activation by autoproteolytic cleavage (35). Then, caspase-11 can directly induce the cleavage of GSDMD to induce pyroptosis (35, 36). Details of the structure and activation mechanism of the NLRP3 inflammasome are refer to following great reviews (10, 17, 39, 40, 52).

## Preeclampsia and the NLRP3 Inflammasome

PE is a pregnancy-specific hypertensive syndrome that complicates around 5–10% of all pregnancies worldwide (53), and is a leading cause of maternal and fetal morbidity and mortality. It is characterized by the onset of hypertension and proteinuria in the third trimester of pregnancy, and is associated with 12% of infants with fetal growth restriction (FGR) and approximately 20% of preterm deliveries (54). The clinical manifestations of PE reflect widespread systemic inflammation and endothelial dysfunction, resulting in vasoconstriction, end-organ ischemia and increased vascular permeability (55). The placenta has been shown to play a central role in the pathogenesis of PE due to the rapid disappearance of disease symptoms after delivery. Thus, placenta-derived circulating factor(s) may induce excessive inflammation and endothelial defects, leading to PE (56).

During normal pregnancy, trophoblast cells invade, and remodeling of maternal spiral arteries and the fetoplacental unit produce angiogenic factors, such as vascular endothelial growth factor (VEGF) and placental growth factor (PlGF), to support the developing placenta (57, 58). Inadequate trophoblast remodeling of spiral arteries, which is a key feature of PE, is believed to result of dysregulation in placental angiogenesis and maternal immune response (55). Following that, various inflammatory factors are produced by the diseased and hypoxic placenta, which activates systemic inflammatory responses (27, 59). It is widely recognized that antiangiogenic factors, including soluble endoglin (sEng; a coreceptor for transforming growth factor β) and soluble fms-like tyrosine kinase (sFlt-1; a receptor for VEGF), induce PE-like phenomena (57, 60). Indeed, overexpression of sEng and sFlt-1 in pregnant rats leads to severe PE symptoms including hypertension, proteinuria, renal and endothelial dysfunction, hemolysis, elevated liver enzymes, and FGR (60).

Pathophysiological changes of PE include inflammation and immune cell activation (61–63). The main pathological features of PE include a general inflammatory response by cytokines, such as IL-1β, IL-6, IL-8, and tumor necrosis factor-α (TNFα) (7, 64, 65). Siljee et al. (66) reported that IL-1β has a potential to improve prediction of PE during the first trimester. A decreased frequency of peripheral Tregs is characteristic immune cell dynamics seen in PE patients (6). On the other hand, M2-like immunomodulatory macrophages are abundantly present in the decidua in healthy pregnant women and participate in spiral artery remodeling via the angiogenic factors, VEGF and PlGF (27). Increased numbers of M1-like inflammatory macrophages are observed in PE patients and may be associated with increase in inflammatory cytokines, decreased spiral artery remodeling, and increased production of sFlt-1 and sEng (27).

**Figure 2:**
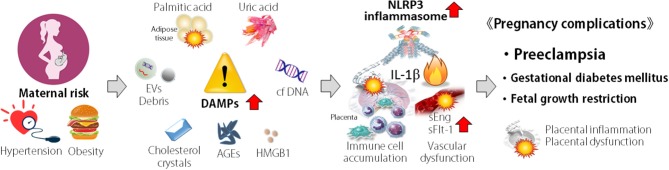
Concept of association of NLRP3 inflammasome in pathogenesis of pregnancy complications. Maternal risk such as hypertension and obesity are associated with the elevation of damage-associated molecular patterns (DAMPs). DAMPs activate NLRP3 inflammasome, accumulate immune cells, and induce inflammatory cytokine production and vascular dysfunction in placenta. These events result in placental inflammation and dysfunction, leading to pregnancy complications, such as preeclampsia, spontaneous abortion, recurrent pregnancy loss, and fetal growth restriction.

In recent years, there has been a rapid increase in reports that the NLRP3 inflammasome is involved in the pathogenesis of PE (Figure 2). Higher expression of components of the NLRP3 inflammasome has been reported in peripheral blood mono-nuclear cells and placental tissue from PE patients compared with that of healthy normal pregnant women (67–69). In addition to immune cells, human trophoblast cells express NLRP3, ASC and caspase-1 that are components of the NLRP3 inflammasome (70–72). IL-1β secretion is induced in response to nigericin or nano-silica crystals, typical activators of the NLRP3 inflammasome, in human trophoblast cells (71, 72).

## Hypertension and the NLRP3 Inflammasome in PE

Maternal hypertension is a characteristic of PE and the renin–angiotensin system has been implicated in its pathogenesis of PE (73, 74) generated a mouse model of PE-like symptoms by mating females expressing human angiotensinogen with males expressing human renin, resulting in mice exhibiting maternal hypertension, proteinuria, and FGR. Angiotensin II (AngII) is a strong vasoconstrictor that contributes to hypertension and stimulates sFlt-1 production and secretion from the placenta in mice (75). Infusion of AngII in pregnant mice can lead to high maternal blood pressure, proteinuria, and FGR (75, 76). Deficiency of NLRP3 inflammasome components attenuates the development of AngII-induced hypertension, but does not affect FGR, proteinuria, or sFlt1 levels (76).

Furthermore, during non-pregnant conditions, infusion of AngII induces hypertension with activation of NLRP3 inflammasome in the aorta, and NLRP3 deficiency attenuated AngII-induced hypertension via inhibition of NLRP3 inflammasome activation in mice (77). A murine experimental hypertension model (uninephrectomy and treatment with deoxycorticosterone acetate and 0.9% NaCl in the drinking water) induced activation of the NLRP3 inflammasome in kidney and specific NLRP3 inhibitor, MCC950, inhibited the NLRP3 inflammasome and inflammation, resulting in improvement of hypertension in mice (78). In rats, salt-induced hypertension occurs partly due to the role of NLRP3 inflammasome activation in the hypothalamic paraventricular nucleus, while blockade of brain NLRP3 attenuates the hypertensive response (79). An absence of ASC also reduces pulmonary hypertension induced by hypoxia (80). These findings suggest that the NLRP3 inflammasome contributes to the development of hypertension in both pregnant and non-pregnant situations. On the other hand, NLRP3 inflammasome has been shown to contribute to a wide range of acute and chronic kidney diseases (81); the importance of NLRP3 inflammasome in renal pathologic abnormalities in PE pathology is not well-understood.

## Activation of NLRP3 Inflammasome by Damps in PE

Release of DAMPs from various cells during stress has been implicated in pregnancy complications. In PE patients, many DAMPs, such as, cholesterol, uric acid crystals (MSU), extracellular DNA, high-mobility group box 1 (HMGB1), extracellular cell debris, advanced glycation end-products (AGEs), and free fatty acids, have been detected in higher levels in the peripheral blood and placenta (Figure 2) and act as NLRP3 inflammasome activators.

## Cholesterol and the NLRP3 inflammasome in PE

Cholesterol crystals activate inflammatory responses and promote inflammatory cell infiltration, resulting in progression of atherosclerosis and development of cardiovascular disease (16, 82). Cholesterol crystals also cause lysosome rupture, resulting in the release of cathepsin B to the cytosol, and are a candidate activator of the NLRP3 inflammasome (82, 83).

Maternal cholesterol serum levels are elevated in PE and cholesterol accumulates in placenta of PE patients, along with increased levels of NLRP3 and IL-1β expression (84, 85). In an *in vitro* human placental explant experiment, treatment with cholesterol crystals significantly increased the release of IL-1β, and cholesterol crystal-induced IL-1β secretion was suppressed by treatment with MCC950, as a specific inhibitor of the NLRP3 inflammasome (84). Cholesterol crystals also strongly activated the NLRP3 inflammasome in macrophages and induced IL-1β secretion, dependent on activation of the NLRP3 inflammasome (82, 83, 86). In addition to macrophages, cholesterol crystals markedly increase the formation and activation of NLRP3 inflammasome in endothelial cells, as demonstrated by increased colocalization of NLRP3 with ASC or caspase-1, enhanced caspase-1 activity, and elevated IL-1β secretion in mice (87). These findings indicate that cholesterol induces placental inflammation via the NLRP3 inflammasome pathway in human placenta, suggesting the contribution of enhanced NLRP3 inflammasome activation to harmful placental inflammation in PE.

## MSU and the NLRP3 Inflammasome in PE

Saturation of uric acid in body fluids results in the formation of MSU crystals. These are identified as danger signals from dying cells, resulting in an acute and/or chronic inflammatory response known as gout, which is associated with the deposition of MSU crystals (41, 88) demonstrated that MSU crystals activate the NLRP3 inflammasome, resulting in the production of active IL-1β and neutrophil accumulation in mice, suggesting a pivotal role for inflammasomes in inflammatory diseases. In terms of the mechanisms of NLRP3 inflammasome activation, MSU crystals are taken up by phagocytosis and lysosomal damage is induced, resulting in the release of cathepsin B and stimulation of ROS production from mitochondria (89).

Elevated levels of MSU in the maternal circulation have been shown in many pregnancy complications, especially PE (69, 84, 90, 91). In human first trimester trophoblast cell lines, IL-1β was produced in response to MSU crystals via the NLRP3 inflammasome (91). Brien et al. (91) reported that MSU crystals induce a proinflammatory profile with predominant secretion of IL-1β and IL-6 in human placental explants, and these effects were IL-1-dependent, as confirmed using a caspase-1 inhibitor and IL-1 receptor antagonist. In addition, administration of MSU crystals to pregnant rats induced placental inflammation (increase IL-1β, IL-6, and TNFα production, and macrophage accumulation) and FGR. Indeed, MSU crystals elicit an increase in the recruitment of macrophages and neutrophils with IL-1β secretion in the NLRP3 inflammasome-dependent manner (41, 92). These findings suggest that higher levels of MSU in PE patients trigger placental inflammation via NLRP3 inflammasome activation, resulting in the pathogenesis of PE.

## Extracellular DNA and the NLRP3 Inflammasome in PE

Extracellular released cell-free DNA (cfDNA) circulating in the blood, which is considered a product of apoptosis and/or necrosis, acts as a DAMP and is related to many types of inflammatory diseases (93, 94). Toll-like receptor 9 (TLR9), originally identified as a sensor of exogenous DNA fragments, contributes to cfDNA-mediated inflammatory processes (95). It is activated by bacterial DNA rich in unmethylated CpG motifs, and can also be activated by DNA from mammalian cells such as nucleic and mitochondrial DNA. Therefore, TLR9 signaling is of interest as a candidate molecule responsible for the first signal in sterile inflammation (96). It was previously reported that cfDNA released from apoptotic hepatocytes activates TLR9 systems, which in turn triggers a signaling cascade that increases transcription of the genes encoding pro-IL-1β and pro-IL-18. Furthermore, mice lacking components of the NLRP3 inflammasome showed reduced amounts of cfDNA and improved liver injury (96). Pan et al., reported that mitochondrial DNA is directly recognized and binds with NLRP3, resulting in the formation of NLRP3 inflammasome complex and its activation (97).

During pregnancy, the amount of total cfDNA and cf-fetal DNA (cffDNA) is significantly increased in the maternal blood depending on the stage of pregnancy (98). There are also significant associations between elevated cfDNA and cffDNA with pregnancy complications such as PE and FGR (98–104). We recently showed that expression levels of TLR9 and the amount of cffDNA from the placenta were higher in PE patients compared with that in women with normal placenta (NP), and PE-derived cffDNA stimulated levels of inflammatory cytokine, including IL-1β and sEng secretion depend on TLR9 signaling, compared with NP-derived cffDNA (105). Moreover, a synthetic TLR9 ligand activated inflammatory responses including IL-6 secretion together with stimulation of sFlt1 secretion, while inhibition of TLR9 reduced sFlt1 secretion in human trophoblast cells (106). In mice, administration of a TLR9 ligand induces PE-like symptoms, such as hypertension, proteinuria, placental inflammation, and FGR. Moreover, injection of human fetal DNA, but not adult DNA, induces placental inflammation, fetal resorption, and preterm birth in pregnant mice, and notably, these adverse effects are improved in TLR9-knockout mice (107). These findings suggest that excessive extracellular DNA acts as a DAMP and causes pregnancy complications, especially PE, via TLR9 signaling.

In trophoblast cells, cfDNA is also capable of detecting danger signals via the intracellular DNA sensor, interferon-inducible protein 16 (IFI16). Indeed, IFI16 agonist poly(dA:dT) stimulates sFlt-1 and sEng production in human trophoblast cells in an IFI16-dependent manner (108). Extracellular DNA plays an essential role in the induction of inflammatory responses; however, further research is required to clarify the role of extracellular DNA in NLRP3 inflammasome activation in pregnancy complications.

## HMGB1 and the NLRP3 Inflammasome in PE

HMGB1 is an important DAMP that acts as an architectural chromatin-binding factor and is generally located in the nucleus of most cell types under physiological conditions (109). When cells are exposed to stress, HMGB1 is translocated into the extracellular milieu and elicits inflammatory responses via the production of proinflammatory mediators and accumulation of inflammatory cells. HMGB1 interacts with TLR2, TLR4, and receptor for AGE (RAGE), resulting in elevated levels of HMGB1 in tissues and serum associated with the development of inflammation during pathological conditions (110). It is reported that HMGB1 induces the formation of the NLRP3 inflammasome (111). HMGB1 also activates the NLRP3 inflammasome since that stimulation with HMGB1 induces the release of IL-1β with increase in NLRP3 inflammasome component, these effects can be attenuated by inhibition of the NLRP3 inflammasome (112). In addition, Deng et al. (113) demonstrated that HMGB1 directly binds LPS and targets its internalization into the lysosomes of cells via the RAGE, resulting activation of caspase-11-dependent non-canonical inflammasome signaling. On the contrary, NLRP3 inflammasome activation accelerates atherosclerosis induced by HMGB1 secretion, indicating that HMGB1 is a key downstream signaling molecule of NLRP3 inflammasome activation (114). Therefore, the vicious cycle of HMGB1 and the NLRP3 inflammasome may exacerbate inflammation and pathological conditions.

In peripheral blood, HMGB1 concentrations are significantly elevated in PE patients compared with those of healthy pregnant and non-pregnant women (115, 116). Compared with healthy placenta, protein and mRNA expression of HMGB1 and its receptor RAGE, are increased in severe PE placentas (116). In human trophoblast cells, HMGB1 stimulates inflammatory cytokine production dependent on NF-κB activation and ROS signaling via TLR4 (117). In human placenta, treatment with PE serum increased the expression and release of HMGB1, which induced endothelial cell activation (118). In addition, HMGB1 treatment increased NLRP3 protein expression and activation of caspase-1, resulting in an increase of mature IL-1β secretion in human chorioamniotic membranes (119). These findings indicated that HMGB1 contributes to placental inflammation and NLRP3 inflammasome activation as endogenous DAMPs, leading to PE. Interestingly, the expression levels of HMGB1 in the uterus are lowest during the expected time of implantation, and exogenous administration of HMGB1 leads to pregnancy failure accompanied by induction of inflammatory responses in rats, indicating a role of excessive extracellular HMGB1 in PE as well as infertility (120).

## Placental Debris and the NLRP3 Inflammasome in PE

The outer layer of the placenta is covered by a single syncytiotrophoblast that forms the maternal-fetal interface (1). When portions of the syncytiotrophoblast become damaged, cellular debris is extruded into the maternal blood in membrane-enclosed vesicles (121). During normal healthy pregnancy, trophoblastic debris is produced by programmed cell death/apoptosis in the placenta. This extracellular debris is believed to induce a tolerogenic response in maternal endothelial and immune cells (122). On the other hand, extracellular debris from PE placenta mainly originates from necrotic cell death, and exposing endothelial cells to necrotic trophoblastic debris leads to their activation (123). The amount of trophoblastic debris shed into the maternal blood is greatly increased in PE patients compared with that in healthy pregnant women (108).

It is likely that trophoblastic debris includes various types of danger signals, such as DNA, RNA, adenosine, HMGB1, and MSU (118, 124). The degree of trophoblastic debris from human placenta is increased by treatment with PE serum and antiphospholipid antibodies, resulting in the activation of endothelial cell activation and induction of immune cell adhesion (118). Interestingly, necrotic, but not apoptotic, trophoblastic debris contains IL-1β protein, whereas much of the trophoblastic debris is dead cell corpses that might not be able to produce new proteins (124). On the other hand, adenosine in trophoblastic debris and cell surface adenosine receptor A2B signaling also contributes to the pathogenesis of PE (125). Iriyama et al. (125) demonstrated that chronically elevated placental adenosine leads to the hallmark features of PE (hypertension, proteinuria, and FGR) in a mouse model. Moreover, elevated adenosine in PE patients is correlated with Th1/Th2 imbalance, and adenosine directly induces sFlt-1 production from placenta (126). Baron et al. (127) showed that extracellular adenosine activates the NLRP3 inflammasome and IL-1β secretion by interaction with adenosine receptors and through adenosine cellular uptake using nucleotide transporters. These findings suggest that adenosine signaling in debris activates NLRP3 inflammasome in placenta, resulting in PE.

## Extracellular Vesicles and the NLRP3 Inflammasome in PE

Extracellular vesicles (EVs) are also produced and released by living cells and can be detected in all biological fluids, including blood. EVs are nanosized particles that are traditionally classified into subtypes, such as exosomes, microvesicles, and apoptotic/necrotic bodies (debris). EV cargo includes bioactive molecules such as protein, lipids, and nucleic acid (DNA, mRNA, microRNA, and non-coding RNA) (128). Significantly higher levels of syncytiotrophoblast-derived EVs are found in the peripheral blood of women with PE compared with women with normal pregnancies (129). EVs isolated from PE patients differ phenotypically and functionally from those isolated from healthy pregnant women (130). Indeed, syncytiotrophoblast-derived EVs (including exosomes) from patients with PE contain higher levels of sFlt-1, sEng, and neprilysin, and treatment with EVs from PE patients impairs angiogenesis of endothelial cells and changes the characteristics of monocytes *in vitro* (131, 132). In addition, exosomes from PE patients cause vascular dysfunction and directly result in adverse PE-like birth outcomes in mice (131). Kohli et al. (133) demonstrated that administration of EVs led to accumulation of activated platelets and induced activation of NLRP3 inflammasome within the placenta, resulting in a PE-like phenotype in pregnant mice. Intriguingly, genetic deletion of NLRP3 inflammasome or pharmacological inhibition of inflammasome abolished this PE-like phenotype, indicating the pathogenesis of PE by EVs was dependent the NLRP3 inflammasome.

## Free Fatty Acid and the NLRP3 Inflammasome in PE

Obesity is a major risk factor for PE and FGR (134, 135). Obesity represents low-grade chronic systemic inflammation (136), and maternal obesity increases the risk of the offspring developing obesity and insulin resistance in the later stages of life (137–141). The NLRP3 inflammasome is involved in the pathogenesis of obesity-related inflammatory diseases, including metabolic syndrome, type 2 diabetes, and cardiovascular diseases (12, 13, 31, 50). There are many common mechanisms between PE and obesity-related pregnancy complications, and obesity accelerates the systemic features of PE.

Free fatty acids levels are elevated in the plasma of obese humans (142), and it has been proposed that they act to promote inflammatory responses by directly engaging TLRs and inducing the NF-κB-dependent production of inflammatory cytokines (143, 144). In particular, one of the major saturated fatty acids, palmitic acid (PA), causes intracellular crystallization, which in turn activates the NLRP3 inflammasome via lysosomal dysfunction in macrophages (145). PA also induces NLRP3 inflammasome activation by generating ROS and inducing autophagy dysfunction, resulting in secretion of mature IL-1β (144, 146, 147). Similar to other crystalline molecules, intraperitoneal administration of PA crystal induces neutrophil recruitment in an IL-1β-dependent manner (145).

Serum PA levels are increased in women with PE and FGR (148–150). Treatment with free fatty acid solution to mimic the plasma of PE patients induces lipid droplet accumulation, mitochondrial dysfunction, and apoptosis in human umbilical vein endothelial cells (149). In addition, PA induces activation of the NLRP3 inflammasome, resulting in the secretion of mature IL-1β by human trophoblast cells (147). NF-κB activation and IL-6 production are associated with higher levels of lipid accumulation in the placenta of obese women compared with those of lean women (151). These findings suggest that saturated fatty acids directly induce placental inflammation, resulting in PE.

## AGEs and the NLRP3 Inflammasome in PE

AGEs are heterogeneous, reactive, and irreversibly crosslinked molecules formed from the non-enzymatic glycation of proteins, lipids, and nucleic acids (152, 153). They interact with RAGE and/or TLR4 to induce inflammatory responses (154, 155). AGE-RAGE interactions may increase and perpetuate the inflammatory condition, leading to obesity, diabetes mellitus, and cardiovascular and kidney diseases. Both *in vivo* and *in vitro* experiments have demonstrated that AGEs stimulate NLRP3 inflammasome activation and IL-1β secretion in human umbilical vein endothelial cells, kidney, and pancreatic islets (117, 156, 157). Ablation of the NLRP3 inflammasome improved AGE-induced abnormal insulin sensitivity, pancreatic islet damage, and inflammatory responses (158). These findings suggest that consumption of AGEs increases obesity-related dysfunction via NLRP3 inflammasome activation.

Increasing evidence indicates that AGEs and IL-1β are associated with PE and obesity in pregnant women (134, 135, 159–161). In human placenta, AGEs increase *in vitro* release of IL-1β, IL-6, IL-8, and TNFα depend on NF-κB activation (162). We also demonstrated that in human placental tissues, AGEs directly increase both the transcription and secretion of IL-1β (117). In addition, AGEs stimulate pro-IL-1β production, resulting in the acceleration of mature IL-1β secretion by NLRP3 inflammasome activation in human trophoblast cells. AGEs also induce sFlt-1 production through RAGE signaling, suggesting a direct link with the pathology of PE (163). Antoniotti et al. (164) reported that AGEs led to activated inflammatory responses in endometrial cells, impaired decidualization, compromised implantation of blastocyst, and suppressed trophoblast invasion. Therefore, AGEs adversely may impact not only PE but also endometrial function and embryo implantation.

## Other Pregnancy Complications Associated with the NLRP3 Inflammasome

GDM is also classed as an obesity-related pregnancy complication. In GDM, high levels of serum glucose are associated with increased inflammation in blood as well as placenta (165). Excess glucose induces IL-1β secretion from human trophoblast cells depending on the NLRP3 inflammasome (166). In addition to the placenta, caspase-1 activation and mature IL-1β secretion are higher in the adipose tissue of pregnant patients with GDM compared with healthy pregnant women (167), and treatment with caspase-1 inhibitor suppresses IL-1β secretion, suggesting the contribution of NLRP3 inflammasome activation in GDM.

Inflammation of the maternal-fetal interface such as intra-amniotic inflammation or chorioamnionitis, which can be induced by intra-amniotic infection or DAMPs, is a causal link to spontaneous preterm birth, which is a leading cause of perinatal mortality and morbidity (168). In a non-primate rhesus macaques chorioamnionitis model induced by intra-amniotic injection of LPS, the amnion upregulated neutrophil accumulation via the chemoattractant IL-8 in an IL-1-dependent manner (169). In a mouse model of intra-amniotic inflammation-induced preterm birth, the NLRP3 inflammasome was activated following IL-1β secretion in the fetal membranes and decidua basalis (170). In addition, IL-1β blockade decreased inflammation-induced preterm labor in mice (171). These findings suggest that the NLRP3 inflammasome plays a pivotal role in inflammation of the maternal-fetal interface associated with preterm birth, and IL-1 is a potential therapeutic target for these conditions.

To understand the role of the NLRP3 inflammasome in normal pregnancy and pregnancy complications, please refer the essential review (172).

## Conclusion

Accumulating evidence suggests that the NLRP3 inflammasome plays an essential role in the pathogenesis of pregnancy inflammatory complications. Various types of DAMPs act as danger signals to activate the NLRP3 inflammasome in reproductive organs, resulting in pregnancy inflammatory complications (Figure 2). Once activated, the NLRP3 inflammasome drives the robust release of mature IL-1β, initiating a positive feedback loop that results in the accumulation of other immune cells (neutrophils and macrophages) and an increase in the “danger” cytokines and chemokines. Considering the potential for excessive NLRP3 inflammasome and IL-1β production, it is not unexpected that several negative regulatory mechanisms exist in nature to control inflammasome function. Understanding how the NLRP3 inflammasome regulates pregnancy complications and how to control excessive NLRP3 inflammasome activation is essential for the identification of new targets for the treatment of reproductive dysfunction.

## Author Contributions

KS and TK wrote the manuscript. MT critically revised the manuscript. All authors read and approved the final manuscript.

### Conflict of Interest

The authors declare that the research was conducted in the absence of any commercial or financial relationships that could be construed as a potential conflict of interest.

## References

[1] Arck PC Hecher K. Fetomaternal immune cross-talk and its consequences for maternal and offspring's health. Nat Med. (2013) 19:548–56. 10.1038/nm.3160 23652115

[2] Medawar P Some immunological and endocrinological problems raised by the evolution of viviparity in vertebrates. Symp Soc Exp Biol. (1952) 7:320–38.

[3] Moffett A Loke C. Immunology of placentation in eutherian mammals. Nat Rev Immunol. (2006) 6:584–94. 10.1038/nri1897 16868549

[4] Akira S Uematsu S Takeuchi O. Pathogen recognition and innate immunity. Cell. (2006) 124:783–801. 10.1016/j.cell.2006.02.015 16497588

[5] Yockey LJ Iwasaki A. Interferons and proinflammatory cytokines in pregnancy and fetal development. Immunity. (2018) 49:397–412. 10.1016/j.immuni.2018.07.017 30231982 PMC6152841

[6] Saito S Nakashima A Ito M Shima T. Clinical implication of recent advances in our understanding of IL-17 and reproductive immunology. Expert Rev Clin Immunol. (2011) 7:649–57. 10.1586/eci.11.49 21895477

[7] Laresgoiti-Servitje E. A leading role for the immune system in the pathophysiology of preeclampsia. J Leukoc Biol. (2013) 94:247–57. 10.1189/jlb.1112603 23633414

[8] Kalagiri RR Carder T Choudhury S Vora N Ballard AR Govande V. Inflammation in complicated pregnancy and its outcome. Am J Perinatol. (2016) 33:1337–56. 10.1055/s-0036-1582397 27159203

[9] Rathinam VA Vanaja SK Fitzgerald KA. Regulation of inflammasome signaling. Nat Immunol. (2012) 13:333–2. 10.1038/ni.2237 22430786 PMC3523703

[10] Strowig T Henao-Mejia J Elinav E Flavell R. Inflammasomes in health and disease. Nature. (2012) 481:278–86. 10.1038/nature10759 22258606

[11] Guo H Callaway JB Ting JP. Inflammasomes: mechanism of action, role in disease, and therapeutics. Nat Med. (2015) 21:677–87. 10.1038/nm.3893 26121197 PMC4519035

[12] Schroder K Zhou R Tschopp J. The NLRP3 inflammasome: a sensor for metabolic danger? Science. (2010) 327:296–300. 10.1126/science.1184003 20075245

[13] Davis BK Wen H Ting JP. The inflammasome NLRs in immunity, inflammation, and associated diseases. Annu Rev Immunol. (2011) 29:707–35. 10.1146/annurev-immunol-031210-101405 21219188 PMC4067317

[14] Usui F Shirasuna K Kimura H Tatsumi K Kawashima A Karasawa T. Critical role of caspase-1 in vascular inflammation and development of atherosclerosis in Western diet-fed apolipoprotein E-deficient mice. Biochem Biophys Res Commun. (2012) 425:162–8. 10.1016/j.bbrc.2012.07.058 22819845

[15] Takahashi M. NLRP3 inflammasome as a novel player in myocardial infarction. Int Heart J. (2014) 55:101–5. 10.1536/ihj.13-388 24632952

[16] Karasawa T Takahashi M. Role of NLRP3 inflammasomes in atherosclerosis. J Atheroscler Thromb. (2017) 24:443–51. 10.5551/jat.RV17001 28260724 PMC5429158

[17] Swanson KV Deng M Ting JP. The NLRP3 inflammasome: molecular activation and regulation to therapeutics. Nat Rev Immunol. (2019) 19:477–89. 10.1038/s41577-019-0165-0 31036962 PMC7807242

[18] Sakaguchi S. Naturally arising CD4+ regulatory t cells for immunologic self-tolerance and negative control of immune responses. Annu Rev Immunol. (2004) 22:531–62. 10.1146/annurev.immunol.21.120601.141122 15032588

[19] Sakaguchi S. Naturally arising Foxp3-expressing CD25+CD4+ regulatory T cells in immunological tolerance to self and non-self. Nat Immunol. (2005) 6:345–52. 10.1038/ni1178 15785760

[20] Sasaki Y Sakai M Miyazaki S Higuma S Shiozaki A Saito S. Decidual and peripheral blood CD4+CD25+ regulatory T cells in early pregnancy subjects and spontaneous abortion cases. Mol Hum Reprod. (2004) 10:347–53. 10.1093/molehr/gah044 14997000

[21] Thuere C Zenclussen ML Schumacher A Langwisch S Schulte-Wrede U Teles A. Kinetics of regulatory T cells during murine pregnancy. Am J Reprod Immunol. (2007) 58:514–23. 10.1111/j.1600-0897.2007.00538.x 17997750

[22] Zhao JX Zeng YY Liu Y. Fetal alloantigen is responsible for the expansion of the CD4(+)CD25(+) regulatory T cell pool during pregnancy. J Reprod Immunol. (2007) 75:71–81. 10.1016/j.jri.2007.06.052 17686527

[23] Shima T Sasaki Y Itoh M Nakashima A Ishii N Sugamura K Regulatory T cells are necessary for implantation and maintenance of early pregnancy but not late pregnancy in allogeneic mice. J Reprod Immunol. (2010) 85:121–9. 10.1016/j.jri.2010.02.006 20439117

[24] Saito S Nakashima A Shima T Ito M. Th1/Th2/Th17 and regulatory T-cell paradigm in pregnancy. Am J Reprod Immunol. (2010) 63:601–10. 10.1111/j.1600-0897.2010.00852.x 20455873

[25] Lee SK Kim JY Lee M Gilman-Sachs A Kwak-Kim J. Th17 and regulatory T cells in women with recurrent pregnancy loss. Am J Reprod Immunol. (2012) 67:311–8. 10.1111/j.1600-0897.2012.01116.x 22380579

[26] Moffett-King A. Natural killer cells and pregnancy. Nat Rev Immunol. (2002) 2:656–63. 10.1038/nri886 12209134

[27] Faas MM Spaans F De Vos P. Monocytes and macrophages in pregnancy and pre-eclampsia. Front Immunol. (2014) 5:298. 10.3389/fimmu.2014.00298 25071761 PMC4074993

[28] Kammerer U Schoppet M McLellan AD Kapp M Huppertz HI Kampgen E. Human decidua contains potent immunostimulatory CD83(+) dendritic cells. Am J Pathol. (2000) 157:159–69. 10.1016/S0002-9440(10)64527-0 10880386 PMC1850207

[29] Blois SM Alba Soto CD Tometten M Klapp BF Margni RA Arck PC. Lineage, maturity, and phenotype of uterine murine dendritic cells throughout gestation indicate a protective role in maintaining pregnancy. Biol Reprod. (2004) 70:1018–23. 10.1095/biolreprod.103.022640 14681197

[30] Steinman RM. Decisions about dendritic cells: past, present, and future. Annu Rev Immunol. (2012) 30:1–22. 10.1146/annurev-immunol-100311-102839 22136168

[31] Takahashi M. Role of the inflammasome in myocardial infarction. Trends Cardiovasc Med. (2011) 21:37–41. 10.1016/j.tcm.2012.02.002 22578238

[32] Gu Y Kuida K Tsutsui H Ku G Hsiao K Fleming MA. Activation of interferon-gamma inducing factor mediated by interleukin-1beta converting enzyme. Science. (1997) 275:206–9. 10.1126/science.275.5297.206 8999548

[33] Lu A Magupalli VG Ruan J Yin Q Atianand MK Vos MR. Unified polymerization mechanism for the assembly of ASC-dependent inflammasomes. Cell. (2014) 156:1193–206. 10.1016/j.cell.2014.02.008 24630722 PMC4000066

[34] Cai X Chen J Xu H Liu S Jiang QX Halfmann R. Prion-like polymerization underlies signal transduction in antiviral immune defense and inflammasome activation. Cell. (2014) 156:1207–22. 10.1016/j.cell.2014.01.063 24630723 PMC4034535

[35] Kayagaki N Stowe IB Lee BL O'Rourke K Anderson K Warming S. Caspase-11 cleaves gasdermin D for non-canonical inflammasome signalling. Nature. (2015) 526:666–71. 10.1038/nature15541 26375259

[36] Shi J Zhao Y Wang K Shi X Wang Y Huang H. Cleavage of GSDMD by inflammatory caspases determines pyroptotic cell death. Nature. (2015) 526:660–5. 10.1038/nature15514 26375003

[37] Juliana C Fernandes-Alnemri T Kang S Farias A Qin F Alnemri ES. Non-transcriptional priming and deubiquitination regulate NLRP3 inflammasome activation. J Biol Chem. (2012) 287:36617–22. 10.1074/jbc.M112.407130 22948162 PMC3476327

[38] Py BF Kim MS Vakifahmetoglu-Norberg H Yuan J. Deubiquitination of NLRP3 by BRCC3 critically regulates inflammasome activity. Mol Cell. (2013) 49:331–8. 10.1016/j.molcel.2012.11.009 23246432

[39] Lamkanfi M Dixit VM. Mechanisms and functions of inflammasomes. Cell. (2014) 157:1013–22. 10.1016/j.cell.2014.04.007 24855941

[40] Vanaja SK Rathinam VA Fitzgerald KA. Mechanisms of inflammasome activation: recent advances and novel insights. Trends Cell Biol. (2015) 25:308–15. 10.1016/j.tcb.2014.12.009 25639489 PMC4409512

[41] Martinon F Petrilli V Mayor A Tardivel A Tschopp J. Gout-associated uric acid crystals activate the NALP3 inflammasome. Nature. (2006) 440:237–41. 10.1038/nature04516 16407889

[42] He Y Zeng MY Yang D Motro B Nunez G. NEK7 is an essential mediator of NLRP3 activation downstream of potassium efflux. Nature. (2016) 530:354–7. 10.1038/nature16959 26814970 PMC4810788

[43] Sharif H Wang L Wang WL Magupalli VG Andreeva L Qiao Q. Structural mechanism for NEK7-licensed activation of NLRP3 inflammasome. Nature. (2019) 570:338–43. 10.1038/s41586-019-1295-z 31189953 PMC6774351

[44] Cassel SL Eisenbarth SC Iyer SS Sadler JJ Colegio OR Tephly LA. The Nalp3 inflammasome is essential for the development of silicosis. Proc Natl Acad Sci USA. (2008) 105:9035–40. 10.1073/pnas.0803933105 18577586 PMC2449360

[45] Hornung V Bauernfeind F Halle A Samstad EO Kono H Rock KL. Silica crystals and aluminum salts activate the NALP3 inflammasome through phagosomal destabilization. Nat Immunol. (2008) 9:847–56. 10.1038/ni.1631 18604214 PMC2834784

[46] Chen S Sun B. Negative regulation of NLRP3 inflammasome signaling. Protein Cell. (2013) 4:251–8. 10.1007/s13238-013-2128-8 23519777 PMC4875520

[47] Karasawa T Kawashima A Usui F Kimura H Shirasuna K Inoue Y. Oligomerized CARD16 promotes caspase-1 assembly and IL-1beta processing. FEBS Open Bio. (2015) 5:348–56. 10.1016/j.fob.2015.04.011 25973362 PMC4420773

[48] Kawashima A Karasawa T Tago K Kimura H Kamata R Usui-Kawanishi F. ARIH2 ubiquitinates NLRP3 and negatively regulates NLRP3 inflammasome activation in macrophages. J Immunol. (2017) 199:3614–22. 10.4049/jimmunol.1700184 29021376

[49] Misawa T Takahama M Kozaki T Lee H Zou J Saitoh T. Microtubule-driven spatial arrangement of mitochondria promotes activation of the NLRP3 inflammasome. Nat Immunol. (2013) 14:454–60. 10.1038/ni.2550 23502856

[50] Chen GY Nunez G. Sterile inflammation: sensing and reacting to damage. Nat Rev Immunol. (2010) 10:826–37. 10.1038/nri2873 21088683 PMC3114424

[51] Shi J Zhao Y Wang Y Gao W Ding J Li P. Inflammatory caspases are innate immune receptors for intracellular LPS. Nature. (2014) 514:187–92. 10.1038/nature13683 25119034

[52] Wen H Miao EA Ting JP. Mechanisms of NOD-like receptor-associated inflammasome activation. Immunity. (2013) 39:432–41. 10.1016/j.immuni.2013.08.037 24054327 PMC3835203

[53] Mol BW Roberts CT Thangaratinam S Magee LA de Groot CJ Hofmeyr GJ. Pre-eclampsia. Lancet. (2015) 387:999–1011. 10.1016/S0140-6736(15)00070-7 26342729

[54] Duley L. The global impact of pre-eclampsia and eclampsia. Semin Perinatol. (2009) 33:130–7. 10.1053/j.semperi.2009.02.010 19464502

[55] Sibai B Dekker G Kupferminc M. Pre-eclampsia. Lancet. (2005) 365:785–99. 10.1016/S0140-6736(05)17987-2 15733721

[56] Roberts JM Taylor RN Musci TJ Rodgers GM Hubel CA McLaughlin MK. Preeclampsia: an endothelial cell disorder. Am J Obstet Gynecol. (1989) 161:1200–4. 10.1016/0002-9378(89)90665-0 2589440

[57] Maynard SE Min JY Merchan J Lim KH Li J Mondal S. Excess placental soluble fms-like tyrosine kinase 1 (sFlt1) may contribute to endothelial dysfunction, hypertension, and proteinuria in preeclampsia. J Clin Invest. (2003) 111:649–58. 10.1172/JCI17189 12618519 PMC151901

[58] Taylor RN Grimwood J Taylor RS McMaster MT Fisher SJ North RA. Longitudinal serum concentrations of placental growth factor: evidence for abnormal placental angiogenesis in pathologic pregnancies. Am J Obstet Gynecol. (2003) 188:177–82. 10.1067/mob.2003.111 12548214

[59] Sacks G Sargent I Redman C. An innate view of human pregnancy. Immunol Today. (1999) 20:114–8. 10.1016/S0167-5699(98)01393-0 10203701

[60] Venkatesha S Toporsian M Lam C Hanai J Mammoto T Kim YM. Soluble endoglin contributes to the pathogenesis of preeclampsia. Nat Med. (2006) 12:642–9. 10.1038/nm1429 16751767

[61] Sacks GP Studena K Sargent K Redman CW. Normal pregnancy and preeclampsia both produce inflammatory changes in peripheral blood leukocytes akin to those of sepsis. Am J Obstet Gynecol. (1998) 179:80–6. 10.1016/S0002-9378(98)70254-6 9704769

[62] Melgert BN Spaans F Borghuis T Klok PA Groen B Bolt A. Pregnancy and preeclampsia affect monocyte subsets in humans and rats. PLoS ONE. (2012) 7:e45229. 10.1371/journal.pone.0045229 23028864 PMC3441708

[63] Lau SY Guild SJ Barrett CJ Chen Q McCowan L Jordan V. Tumor necrosis factor-alpha, interleukin-6, and interleukin-10 levels are altered in preeclampsia: a systematic review and meta-analysis. Am J Reprod Immunol. (2013) 70:412–27. 10.1111/aji.12138 23790133

[64] Mellembakken JR Aukrust P Hestdal K Ueland T Abyholm T Videm V. Chemokines and leukocyte activation in the fetal circulation during preeclampsia. Hypertension. (2001) 38:394–8. 10.1161/01.HYP.38.3.394 11566911

[65] Mor G Cardenas I Abrahams V Guller S. Inflammation and pregnancy: the role of the immune system at the implantation site. Ann N Y Acad Sci. (2011) 1221:80–7. 10.1111/j.1749-6632.2010.05938.x 21401634 PMC3078586

[66] Siljee JE Wortelboer EJ Koster MP Imholz S Rodenburg W Visser GH. Identification of interleukin-1 beta, but no other inflammatory proteins, as an early onset pre-eclampsia biomarker in first trimester serum by bead-based multiplexed immunoassays. Prenat Diagn. (2013) 33:1183–8. 10.1002/pd.4219 23943085

[67] Xie F Hu Y Turvey SE Magee LA Brunham RM Choi KC. Toll-like receptors 2 and 4 and the cryopyrin inflammasome in normal pregnancy and pre-eclampsia. BJOG. (2010) 117:99–108. 10.1111/j.1471-0528.2009.02428.x 20002372

[68] Matias ML Romao M Weel IC Ribeiro VR Nunes PR Borges VT. Endogenous and uric acid-induced activation of NLRP3 inflammasome in pregnant women with preeclampsia. PLoS ONE. (2015) 10:e0129095. 10.1371/journal.pone.0129095 26053021 PMC4459873

[69] C Weel I Romao-Veiga M Matias ML Fioratti EG Peracoli JC Borges VT. Increased expression of NLRP3 inflammasome in placentas from pregnant women with severe preeclampsia. J Reprod Immunol. (2017) 123:40–7. 10.1016/j.jri.2017.09.002 28915449

[70] Mulla MJ Myrtolli K Potter J Boeras C Kavathas PB Sfakianaki AK. Uric acid induces trophoblast IL-1beta production via the inflammasome: implications for the pathogenesis of preeclampsia. Am J Reprod Immunol. (2011) 65:542–8. 10.1111/j.1600-0897.2010.00960.x 21352397 PMC3114103

[71] Shirasuna K Usui F Karasawa T Kimura H Kawashima A Mizukami H. Nanosilica-induced placental inflammation and pregnancy complications: different roles of the inflammasome components NLRP3 and ASC. Nanotoxicology. (2015) 9:554–67. 10.3109/17435390.2014.956156 25211550

[72] Tamura K Ishikawa G Yoshie M Ohneda W Nakai A Takeshita T. Glibenclamide inhibits NLRP3 inflammasome-mediated IL-1beta secretion in human trophoblasts. J Pharmacol Sci. (2017) 135:89–95. 10.1016/j.jphs.2017.09.032 29056256

[73] Furuya M Ishida J Aoki I Fukamizu A. Pathophysiology of placentation abnormalities in pregnancy-induced hypertension. Vasc Health Risk Manag. (2008) 4:1301–13. 10.2147/VHRM.S4009 19337544 PMC2663465

[74] Takimoto E Ishida J Sugiyama F Horiguchi H Murakami K Fukamizu A. Hypertension induced in pregnant mice by placental renin and maternal angiotensinogen. Science. (1996) 274:995–8. 10.1126/science.274.5289.995 8875944

[75] Zhou CC Ahmad S Mi T Xia L Abbasi S Hewett PW. Angiotensin II induces soluble fms-Like tyrosine kinase-1 release via calcineurin signaling pathway in pregnancy. Circ Res. (2007) 100:88–95. 10.1161/01.RES.0000254703.11154.18 17158338 PMC3266823

[76] Shirasuna K Karasawa T Usui F Kobayashi M Komada T Kimura H NLRP3 Deficiency improves angiotensin II-induced hypertension but not fetal growth restriction during pregnancy. Endocrinology. (2015) 156:4281–92. 10.1210/en.2015-1408 26360504

[77] Ren XS Tong Y Ling L Chen D Sun HJ Zhou H. NLRP3 gene deletion attenuates angiotensin ii-induced phenotypic transformation of vascular smooth muscle cells and vascular remodeling. Cell Physiol Biochem. (2017) 44:2269–80. 10.1159/000486061 29262411

[78] Krishnan SM Ling YH Huuskes BM Ferens DM Saini N Chan CT. Pharmacological inhibition of the NLRP3 inflammasome reduces blood pressure, renal damage, and dysfunction in salt-sensitive hypertension. Cardiovasc Res. (2019) 115:776–87. 10.1093/cvr/cvy252 30357309 PMC6432065

[79] Wang ML Kang YM Li XG Su Q Li HB Liu KL. Central blockade of NLRP3 reduces blood pressure via regulating inflammation microenvironment and neurohormonal excitation in salt-induced prehypertensive rats. J Neuroinflam. (2018) 15:95. 10.1186/s12974-018-1131-7 29573749 PMC5866519

[80] Cero FT Hillestad V Sjaastad I Yndestad A Aukrust P Ranheim T. Absence of the inflammasome adaptor ASC reduces hypoxia-induced pulmonary hypertension in mice. Am J Physiol Lung Cell Mol Physiol. (2015) 309:L378–87. 10.1152/ajplung.00342.2014 26071556

[81] Komada T Muruve DA. The role of inflammasomes in kidney disease. Nat Rev Nephrol. (2019) 15:501–20. 10.1038/s41581-019-0158-z 31164720

[82] Duewell P Kono H Rayner KJ Sirois CM Vladimer G Bauernfeind FG. NLRP3 inflammasomes are required for atherogenesis and activated by cholesterol crystals. Nature. (2010) 464:1357–61. 10.1038/nature08938 20428172 PMC2946640

[83] Freigang S Ampenberger F Spohn G Heer S Shamshiev AT Kisielow J. Nrf2 is essential for cholesterol crystal-induced inflammasome activation and exacerbation of atherosclerosis. Eur J Immunol. (2011) 41:2040–51. 10.1002/eji.201041316 21484785

[84] Stodle GS Silva GB Tangeras LH Gierman LM Nervik I Dahlberg UE. Placental inflammation in pre-eclampsia by Nod-like receptor protein (NLRP)3 inflammasome activation in trophoblasts. Clin Exp Immunol. (2018) 193:84–94. 10.1111/cei.13130 29683202 PMC6038006

[85] Jabalie G Ahmadi M Koushaeian L Eghbal-Fard S Mehdizadeh A Kamrani A. Metabolic syndrome mediates proinflammatory responses of inflammatory cells in preeclampsia. Am J Reprod Immunol. (2019) 81:e13086. 10.1111/aji.13086 30614120

[86] Liu W Yin Y Zhou Z He M Dai Y. OxLDL-induced IL-1 beta secretion promoting foam cells formation was mainly via CD36 mediated ROS production leading to NLRP3 inflammasome activation. Inflamm Res. (2014) 63:33–43. 10.1007/s00011-013-0667-3 24121974

[87] Koka S Xia M Chen Y Bhat OM Yuan X Boini KM. Endothelial NLRP3 inflammasome activation and arterial neointima formation associated with acid sphingomyelinase during hypercholesterolemia. Redox Biol. (2017) 13:336–44. 10.1016/j.redox.2017.06.004 28633109 PMC5479959

[88] Shi Y Evans JE Rock KL. Molecular identification of a danger signal that alerts the immune system to dying cells. Nature. (2003) 425:516–21. 10.1038/nature01991 14520412

[89] Gross O Yazdi AS Thomas CJ Masin M Heinz LX Guarda G. Inflammasome activators induce interleukin-1alpha secretion via distinct pathways with differential requirement for the protease function of caspase-1. Immunity. (2012) 36:388–400. 10.1016/j.immuni.2012.01.018 22444631

[90] Girard S Heazell AE Derricott H Allan SM Sibley CP Abrahams VM. Circulating cytokines and alarmins associated with placental inflammation in high-risk pregnancies. Am J Reprod Immunol. (2014) 72:422–34. 10.1111/aji.12274 24867252 PMC4369138

[91] Brien ME Duval C Palacios J Boufaied I Hudon-Thibeault AA Nadeau-Vallee M. Uric acid crystals induce placental inflammation and alter trophoblast function via an IL-1-dependent pathway: implications for fetal growth restriction. J Immunol. (2017) 198:443–51. 10.4049/jimmunol.1601179 27903743 PMC5176081

[92] Mitroulis I Kambas K Ritis K. Neutrophils, IL-1beta, and gout: is there a link? Semin Immunopathol. (2013) 35:501–12. 10.1007/s00281-013-0361-0 23344781

[93] Atamaniuk J Kopecky C Skoupy S Saemann MD Weichhart T. Apoptotic cell-free DNA promotes inflammation in haemodialysis patients. Nephrol Dial Transplant. (2012) 27:902–5. 10.1093/ndt/gfr695 22167588

[94] Nishimoto S Fukuda D Higashikuni Y Tanaka K Hirata Y Murata C. Obesity-induced DNA released from adipocytes stimulates chronic adipose tissue inflammation and insulin resistance. Sci Adv. (2016) 2:e1501332. 10.1126/sciadv.1501332 27051864 PMC4820373

[95] Vollmer J. TLR9 in health and disease. Int Rev Immunol. (2006) 25:155–81. 10.1080/08830180600743107 16818370

[96] Imaeda AB Watanabe A Sohail MA Mahmood S Mohamadnejad M Sutterwala FS. Acetaminophen-induced hepatotoxicity in mice is dependent on Tlr9 and the Nalp3 inflammasome. J Clin Invest. (2009) 119:305–14. 10.1172/JCI35958 19164858 PMC2631294

[97] Pan J Ou Z Cai C Li P Gong J Ruan XZ. Fatty acid activates NLRP3 inflammasomes in mouse kupffer cells through mitochondrial DNA release. Cell Immunol. (2018) 332:111–20. 10.1016/j.cellimm.2018.08.006 30103942

[98] Sur Chowdhury C Hahn S Hasler P Hoesli I Lapaire O Giaglis S. Elevated levels of total cell-free DNA in maternal serum samples arise from the generation of neutrophil extracellular traps. Fetal Diagn Ther. (2016) 40:263–7. 10.1159/000444853 26998969

[99] Martin A Krishna I Badell M Samuel A. Can the quantity of cell-free fetal DNA predict preeclampsia: a systematic review. Prenat Diagn. (2014) 34:685–91. 10.1002/pd.4416 24852111

[100] Taglauer ES Wilkins-Haug L Bianchi DW. Review: cell-free fetal DNA in the maternal circulation as an indication of placental health and disease. Placenta. (2014) 35:S64–8. 10.1016/j.placenta.2013.11.014 24388429 PMC4886648

[101] Eche S Mackraj I Moodley J. Circulating fetal and total cell-free DNA, and sHLA-G in black South African women with gestational hypertension and pre-eclampsia. Hypert Pregna. (2017) 36:295–301. 10.1080/10641955.2017.1385794 29115889

[102] Munoz-Hernandez R Medrano-Campillo P Miranda ML Macher HC Praena-Fernandez JM Vallejo-Vaz AJ. Total and fetal circulating cell-free DNA, angiogenic, and antiangiogenic factors in preeclampsia and HELLP syndrome. Am J Hypertens. (2017) 30:673–82. 10.1093/ajh/hpx024 28338787

[103] Konecna B Laukova L Vlkova B. Immune activation by nucleic acids: a role in pregnancy complications. Scand J Immunol. (2018) 87:e12651. 10.1111/sji.12651 29479732

[104] van Boeckel SR Davidson DJ Norman JE Stock SJ. Cell-free fetal DNA and spontaneous preterm birth. Reproduction. (2018) 155:R137–45. 10.1530/REP-17-0619 29269517 PMC5812054

[105] Ozeki A Tani K Takahashi H Suzuki H Nagayama S Hirashima C. Preeclamptic patient-derived circulating cell-free DNA activates the production of inflammatory cytokines via toll-like receptor 9 signalling in the human placenta. J Hypertens. (2019) 37:2452–60. 10.1097/HJH.0000000000002208 31385869

[106] He B Yang X Li Y Huang D Xu X Yang W. TLR9 (Toll-like receptor 9) agonist suppresses angiogenesis by differentially regulating VEGFA (vascular endothelial growth factor A) and sFLT1 (soluble vascular endothelial growth factor receptor 1) in preeclampsia. Hypertension. (2018) 71:671–80. 10.1161/HYPERTENSIONAHA.117.10510 29437897

[107] Scharfe-Nugent A Corr SC Carpenter SB Keogh L Doyle B Martin C. TLR9 provokes inflammation in response to fetal DNA: mechanism for fetal loss in preterm birth and preeclampsia. J Immunol. (2012) 188:5706–12. 10.4049/jimmunol.1103454 22544937

[108] Li N Fu Y Chen W Hu GQ Zhou M Yu SX. IFI16 mediates soluble Flt-1 and endoglin production by trophoblast cells. J Hypertens. (2015) 33:1658–65. 10.1097/HJH.0000000000000605 26002845

[109] Andersson U Tracey KJ. HMGB1 as a mediator of necrosis-induced inflammation and a therapeutic target in arthritis. Rheum Dis Clin North Am. (2004) 30:627–37. 10.1016/j.rdc.2004.04.007 15261345

[110] Pisetsky DS Erlandsson-Harris H Andersson U. High-mobility group box protein 1 (HMGB1): an alarmin mediating the pathogenesis of rheumatic disease. Arthritis Res Ther. (2008) 10:209. 10.1186/ar2440 18598385 PMC2483460

[111] Yao X Jiang Q Ding W Yue P Wang J Zhao K. Interleukin 4 inhibits high mobility group box-1 protein-mediated NLRP3 inflammasome formation by activating peroxisome proliferator-activated receptor-gamma in astrocytes. Biochem Biophys Res Commun. (2019) 509:624–31. 10.1016/j.bbrc.2018.11.145 30606476

[112] Kim EJ Park SY Baek SE Jang MA Lee WS Bae SS. HMGB1 increases IL-1beta production in vascular smooth muscle cells via NLRP3 inflammasome. Front Physiol. (2018) 9:313. 10.3389/fphys.2018.00313 29643819 PMC5882820

[113] Deng M Tang Y Li W Wang X Zhang R Zhang X. The endotoxin delivery protein HMGB1 mediates caspase-11-dependent lethality in sepsis. Immunity. (2018) 49:740–53 e747. 10.1016/j.immuni.2018.08.016 30314759 PMC6300139

[114] Wang R Wu W Li W Huang S Li Z Liu R. Activation of NLRP3 inflammasome promotes foam cell formation in vascular smooth muscle cells and atherogenesis via HMGB1. J Am Heart Assoc. (2018) 7:e008596. 10.1161/JAHA.118.008596 30371306 PMC6404867

[115] Pradervand PA Clerc S Frantz J Rotaru C Bardy D Waeber B. High mobility group box 1 protein (HMGB-1): a pathogenic role in preeclampsia? Placenta. (2014) 35:784–6. 10.1016/j.placenta.2014.06.370 25043672

[116] Zhu L Zhang Z Zhang L Shi Y Qi J Chang A. HMGB1-RAGE signaling pathway in severe preeclampsia. Placenta. (2015) 36:1148–52. 10.1016/j.placenta.2015.08.006 26303759

[117] Seno K Sase S Ozeki A Takahashi H Ohkuchi A Suzuki H. Advanced glycation end products regulate interleukin-1beta production in human placenta. J Reprod Dev. (2017) 63:401–8. 10.1262/jrd.2017-032 28515391 PMC5593091

[118] Shao J Zhao M Tong M Wei J Wise MR Stone P. Increased levels of HMGB1 in trophoblastic debris may contribute to preeclampsia. Reproduction. (2016) 152:775–84. 10.1530/REP-16-0083 27658754

[119] Plazyo O Romero R Unkel R Balancio A Mial TN Xu Y. HMGB1 induces an inflammatory response in the chorioamniotic membranes that is partially mediated by the inflammasome. Biol Reprod. (2016) 95:130. 10.1095/biolreprod.116.144139 27806943 PMC5315428

[120] Bhutada S Basak T Savardekar L Katkam RR Jadhav G Metkari SM. High mobility group box 1 (HMGB1) protein in human uterine fluid and its relevance in implantation. Hum Reprod. (2014) 29:763–80. 10.1093/humrep/det461 24488797

[121] Coleman SJ Gerza L Jones CJ Sibley CP Aplin JD Heazell AE. Syncytial nuclear aggregates in normal placenta show increased nuclear condensation, but apoptosis and cytoskeletal redistribution are uncommon. Placenta. (2013) 34:449–55. 10.1016/j.placenta.2013.02.007 23507147 PMC3661987

[122] Chen Q Guo F Jin HY Lau S Stone P Chamley L. Phagocytosis of apoptotic trophoblastic debris protects endothelial cells against activation. Placenta. (2012) 33:548–53. 10.1016/j.placenta.2012.03.007 22504042

[123] Chen Q Stone PR McCowan LM Chamley LW Phagocytosis of necrotic but not apoptotic trophoblasts induces endothelial cell activation. Hypertension. (2006) 47:116–21. 10.1161/01.HYP.0000196731.56062.7c 16344369

[124] Wei J Chen Q James JL Stone PR Chamley LW. IL-1 beta but not the NALP3 inflammasome is an important determinant of endothelial cell responses to necrotic/dangerous trophoblastic debris. Placenta. (2015) 36:1385–92. 10.1016/j.placenta.2015.10.011 26515928

[125] Iriyama T Sun K Parchim NF Li J Zhao C Song A. Elevated placental adenosine signaling contributes to the pathogenesis of preeclampsia. Circulation. (2015) 131:730–41. 10.1161/CIRCULATIONAHA.114.013740 25538227 PMC4751998

[126] George EM Cockrell K Adair TH Granger JP. Regulation of sFlt-1 and VEGF secretion by adenosine under hypoxic conditions in rat placental villous explants. Am J Physiol Regul Integr Comp Physiol. (2010) 299:R1629–33. 10.1152/ajpregu.00330.2010 20962204 PMC3007189

[127] Baron L Gombault A Fanny M Villeret B Savigny F Guillou N. The NLRP3 inflammasome is activated by nanoparticles through ATP, ADP and adenosine. Cell Death Dis. (2015) 6:e1629. 10.1038/cddis.2014.576 25654762 PMC4669808

[128] Chiarello DI Salsoso R Toledo F Mate A Vazquez CM Sobrevia L. Foetoplacental communication via extracellular vesicles in normal pregnancy and preeclampsia. Mol Aspects Med. (2018) 60:69–80. 10.1016/j.mam.2017.12.002 29222068

[129] Knight M Redman CW Linton EA Sargent IL. Shedding of syncytiotrophoblast microvilli into the maternal circulation in pre-eclamptic pregnancies. Br J Obstet Gynaecol. (1998) 105:632–40. 10.1111/j.1471-0528.1998.tb10178.x 9647154

[130] Boisrame-Helms J Meziani F Sananes N Boisrame T Langer B Schneider F. Detrimental arterial inflammatory effect of microparticles circulating in preeclamptic women: *ex vivo* evaluation in human arteries. Fundam Clin Pharmacol. (2015) 29:450–61. 10.1111/fcp.12136 26213341

[131] Chang X Yao J He Q Liu M Duan T Wang K. Exosomes from women with preeclampsia induced vascular dysfunction by delivering sFlt (soluble fms-like tyrosine kinase)-1 and sEng (soluble endoglin) to endothelial cells. Hypertension. (2018) 72:1381–90. 10.1161/HYPERTENSIONAHA.118.11706 30571229

[132] Gill M Motta-Mejia C Kandzija N Cooke W Zhang W Cerdeira AS. Placental syncytiotrophoblast-derived extracellular vesicles carry active NEP (neprilysin) and are increased in preeclampsia. Hypertension. (2019) 73:1112–9. 10.1161/HYPERTENSIONAHA.119.12707 30929513

[133] Kohli S Ranjan S Hoffmann J Kashif M Daniel EA Al-Dabet MM. Maternal extracellular vesicles and platelets promote preeclampsia via inflammasome activation in trophoblasts. Blood. (2016) 128:2153–64. 10.1182/blood-2016-03-705434 27589872

[134] Catalano PM Ehrenberg HM. The short- and long-term implications of maternal obesity on the mother and her offspring. BJOG. (2006) 113:1126–33. 10.1111/j.1471-0528.2006.00989.x 16827826

[135] Marchi J Berg M Dencker A Olander EK Begley C. Risks associated with obesity in pregnancy, for the mother and baby: a systematic review of reviews. Obes Rev. (2015) 16:621–38. 10.1111/obr.12288 26016557

[136] Prieto D Contreras C Sanchez A. Endothelial dysfunction, obesity and insulin resistance. Curr Vasc Pharmacol. (2014) 12:412–26. 10.2174/1570161112666140423221008 24846231

[137] Challier JC Basu S Bintein T Minium J Hotmire K Catalano PM. Obesity in pregnancy stimulates macrophage accumulation and inflammation in the placenta. Placenta. (2008) 29:274–81. 10.1016/j.placenta.2007.12.010 18262644 PMC4284075

[138] Jungheim ES Schoeller EL Marquard KL Louden ED Schaffer JE Moley KH. Diet-induced obesity model: abnormal oocytes and persistent growth abnormalities in the offspring. Endocrinology. (2010) 151:4039–46. 10.1210/en.2010-0098 20573727 PMC2940512

[139] Jungheim ES Louden ED Chi MM Frolova AI Riley JK Moley KH. Preimplantation exposure of mouse embryos to palmitic acid results in fetal growth restriction followed by catch-up growth in the offspring. Biol Reprod. (2011) 85:678–83. 10.1095/biolreprod.111.092148 21653893 PMC3184288

[140] Shankar K Zhong Y Kang P Lau F Blackburn ML Chen JR. Maternal obesity promotes a proinflammatory signature in rat uterus and blastocyst. Endocrinology. (2011) 152:4158–70. 10.1210/en.2010-1078 21862610 PMC3199010

[141] Aye IL Jansson T Powell TL. Interleukin-1beta inhibits insulin signaling and prevents insulin-stimulated system A amino acid transport in primary human trophoblasts. Mol Cell Endocrinol. (2013) 381:46–55. 10.1016/j.mce.2013.07.013 23891856 PMC3795822

[142] Boden G. Interaction between free fatty acids and glucose metabolism. Curr Opin Clin Nutr Metab Care. (2002) 5:545–9. 10.1097/00075197-200209000-00014 12172479

[143] Shi H Kokoeva MV Inouye K Tzameli I Yin H Flier JS. TLR4 links innate immunity and fatty acid-induced insulin resistance. J Clin Invest. (2006) 116:3015–25. 10.1172/JCI28898 17053832 PMC1616196

[144] Wen H Gris D Lei Y Jha S Zhang L Huang MT. Fatty acid-induced NLRP3-ASC inflammasome activation interferes with insulin signaling. Nat Immunol. (2011) 12:408–15. 10.1038/ni.2022 21478880 PMC4090391

[145] Karasawa T Kawashima A Usui-Kawanishi F Watanabe S Kimura H Kamata R. Saturated fatty acids undergo intracellular crystallization and activate the NLRP3 inflammasome in macrophages. Arterioscler Thromb Vasc Biol. (2018) 38:744–56. 10.1161/ATVBAHA.117.310581 29437575

[146] L'Homme L Esser N Riva L Scheen A Paquot N Piette J. Unsaturated fatty acids prevent activation of NLRP3 inflammasome in human monocytes/macrophages. J Lipid Res. (2013) 54:2998–3008. 10.1194/jlr.M037861 24006511 PMC3793604

[147] Shirasuna K Takano H Seno K Ohtsu A Karasawa T Takahashi M. Palmitic acid induces interleukin-1beta secretion via NLRP3 inflammasomes and inflammatory responses through ROS production in human placental cells. J Reprod Immunol. (2016) 116:104–12. 10.1016/j.jri.2016.06.001 27300134

[148] Endresen MJ Tosti E Heimli H Lorentzen B Henriksen T. Effects of free fatty acids found increased in women who develop pre-eclampsia on the ability of endothelial cells to produce prostacyclin, cGMP and inhibit platelet aggregation. Scand J Clin Lab Invest. (1994) 54:549–57. 10.3109/00365519409088567 7863232

[149] Robinson NJ Minchell LJ Myers JE Hubel CA Crocker IP. A potential role for free fatty acids in the pathogenesis of preeclampsia. J Hypertens. (2009) 27:1293–302. 10.1097/HJH.0b013e328329fbfe 19462499

[150] Ortega-Senovilla H Alvino G Taricco E Cetin I Herrera E. Enhanced circulating retinol and non-esterified fatty acids in pregnancies complicated with intrauterine growth restriction. Clin Sci. (2010) 118:351–8. 10.1042/CS20090292 19656084

[151] Saben J Lindsey F Zhong Y Thakali K Badger TM Andres A. Maternal obesity is associated with a lipotoxic placental environment. Placenta. (2014) 35:171–7. 10.1016/j.placenta.2014.01.003 24484739 PMC3978121

[152] Brownlee M Cerami A Vlassara H. Advanced glycosylation end products in tissue and the biochemical basis of diabetic complications. N Engl J Med. (1988) 318:1315–21. 10.1056/NEJM198805193182007 3283558

[153] John WG Lamb EJ The maillard or browning reaction in diabetes. Eye. (1993) 7:230–7. 10.1038/eye.1993.55 7607341

[154] Ibrahim ZA Armour CL Phipps S Sukkar MB RAGE and TLRs: relatives, friends or neighbours? Mol Immunol. (2013) 56:739–44. 10.1016/j.molimm.2013.07.008 23954397

[155] Shirasuna K Seno K Ohtsu A Shiratsuki S Ohkuchi A Suzuki H. AGEs and HMGB1 increase inflammatory cytokine production from human placental cells, resulting in an enhancement of monocyte migration. Am J Reprod Immunol. (2016) 75:557–68. 10.1111/aji.12506 26961863

[156] Yeh WJ Yang HY Pai MH Wu CH Chen JR. Long-term administration of advanced glycation end-product stimulates the activation of NLRP3 inflammasome and sparking the development of renal injury. J Nutr Biochem. (2017) 39:68–76. 10.1016/j.jnutbio.2016.09.014 27816762

[157] Cao X Xia Y Zeng M Wang W He Y Liu J. Caffeic acid inhibits the formation of advanced glycation end products (AGEs) and mitigates the AGEs-induced the oxidative stress and inflammation reaction in human umbilical vein endothelial cells (HUVECs). Chem Biodivers. (2019) 16:e1900174. 10.1002/cbdv.201900174 31419039

[158] Kong X Lu AL Yao XM Hua Q Li XY Qin L. Activation of NLRP3 inflammasome by advanced glycation end products promotes pancreatic islet damage. Oxid Med Cell Longev. (2017) 2017:9692546. 10.1155/2017/9692546 29230270 PMC5694574

[159] Chekir C Nakatsuka M Noguchi S Konishi H Kamada Y Sasaki A. Accumulation of advanced glycation end products in women with preeclampsia: possible involvement of placental oxidative and nitrative stress. Placenta. (2006) 27:225–33. 10.1016/j.placenta.2005.02.016 16338468

[160] Alexander KL Mejia CA Jordan C Nelson MB Howell BM Jones CM. Differential receptor for advanced glycation end products expression in preeclamptic, intrauterine growth restricted, and gestational diabetic placentas. Am J Reprod Immunol. (2016) 75:172–80. 10.1111/aji.12462 26682535

[161] Chen W Zhang Y Yue C Ye Y Chen P Peng W. Accumulation of advanced glycation end products involved in inflammation and contributing to severe preeclampsia, in maternal blood, umbilical blood and placental tissues. Gynecol Obstet Invest. (2016) 82:388–97. 10.1159/000448141 27505171

[162] Lappas M Permezel M Rice GE. Advanced glycation endproducts mediate pro-inflammatory actions in human gestational tissues via nuclear factor-kappaB and extracellular signal-regulated kinase 1/2. J Endocrinol. (2007) 193:269–77. 10.1677/JOE-06-0081 17470518

[163] Huang QT Zhang M Zhong M Yu YH Liang WZ Hang LL. Advanced glycation end products as an upstream molecule triggers ROS-induced sFlt-1 production in extravillous trophoblasts: a novel bridge between oxidative stress and preeclampsia. Placenta. (2013) 34:1177–82. 10.1016/j.placenta.2013.09.017 24144948

[164] Antoniotti GS Coughlan M Salamonsen LA Evans J. Obesity associated advanced glycation end products within the human uterine cavity adversely impact endometrial function and embryo implantation competence. Hum Reprod. (2018) 33:654–65. 10.1093/humrep/dey029 29471449

[165] Corrêa-Silva S Alencar AP Moreli JB Borbely AU de S Lima L Scavone C. Hyperglycemia induces inflammatory mediators in the human chorionic villous. Cytokine. (2018) 111:41–8. 10.1016/j.cyto.2018.07.020 30114628

[166] Han CS Herrin MA Pitruzzello MC Mulla MJ Werner EF Pettker CM. Glucose and metformin modulate human first trimester trophoblast function: a model and potential therapy for diabetes-associated uteroplacental insufficiency. Am J Reprod Immunol. (2015) 73:362–71. 10.1111/aji.12339 25394884 PMC4356646

[167] Lappas M. Activation of inflammasomes in adipose tissue of women with gestational diabetes. Mol Cell Endocrinol. (2014) 382:74–83. 10.1016/j.mce.2013.09.011 24055273

[168] Nadeau-Vallee M Obari D Palacios J Brien ME Duval C Chemtob S. Sterile inflammation and pregnancy complications: a review. Reproduction. (2016) 152:R277–92. 10.1530/REP-16-0453 27679863

[169] Presicce P Park CW Senthamaraikannan P Bhattacharyya S Jackson C Kong F. IL-1 signaling mediates intrauterine inflammation and chorio-decidua neutrophil recruitment and activation. JCI Insight. (2018) 3:98306. 10.1172/jci.insight.98306 29563340 PMC5926925

[170] Faro J Romero R Schwenkel G Garcia-Flores V Arenas-Hernandez M Leng Y. Intra-amniotic inflammation induces preterm birth by activating the NLRP3 inflammasomedagger. Biol Reprod. (2019) 100:1290–305. 10.1093/biolre/ioy261 30590393 PMC6698670

[171] Nadeau-Vallee M Quiniou C Palacios J Hou X Erfani A Madaan A. Novel noncompetitive IL-1 receptor-biased ligand prevents infection- and inflammation-induced preterm birth. J Immunol. (2015) 195:3402–15. 10.4049/jimmunol.1500758 26304990

[172] Gomez-Lopez N Motomura K Miller D Garcia-Flores V Galaz J Romero R. Inflammasomes: their role in normal and complicated pregnancies. J Immunol. (2019) 203:2757–69. 10.4049/jimmunol.1900901 31740550 PMC6871659

